# The Effect of Rural-to-Urban Migration on Obesity and Diabetes in India: A Cross-Sectional Study

**DOI:** 10.1371/journal.pmed.1000268

**Published:** 2010-04-27

**Authors:** Shah Ebrahim, Sanjay Kinra, Liza Bowen, Elizabeth Andersen, Yoav Ben-Shlomo, Tanica Lyngdoh, Lakshmy Ramakrishnan, R. C. Ahuja, Prashant Joshi, S. Mohan Das, Murali Mohan, George Davey Smith, Dorairaj Prabhakaran, K. Srinath Reddy, for the Indian Migration Study group

**Affiliations:** 1 Department of Epidemiology & Population Health, London School of Hygiene & Tropical Medicine, London, United Kingdom; 2 South Asia Network for Chronic Disease, Public Health Foundation of India, New Delhi, India; 3 Department of Social Medicine, University of Bristol, Bristol, United Kingdom; 4 Department of Biochemistry, All India Institute of Medical Sciences, New Delhi, India; 5 Department of Medicine, King George's Medical College and Institute of Clinical Epidemiology, Lucknow, India; 6 Department of Medicine, Government Medical College, Nagpur, India; 7 Department of Neurology, Krishna Institute of Medical Sciences, Hyderabad, India; 8 Department of Medicine, Dr. B R Ambedkar Medical College, Bangalore, India; 9 Centre for Chronic Disease Control, New Delhi, India; 10 Public Health Foundation of India, New Delhi, India

## Abstract

**Background:**

Migration from rural areas of India contributes to urbanisation and may increase the risk of obesity and diabetes. We tested the hypotheses that rural-to-urban migrants have a higher prevalence of obesity and diabetes than rural nonmigrants, that migrants would have an intermediate prevalence of obesity and diabetes compared with life-long urban and rural dwellers, and that longer time since migration would be associated with a higher prevalence of obesity and of diabetes.

**Methods and Findings:**

The place of origin of people working in factories in north, central, and south India was identified. Migrants of rural origin, their rural dwelling sibs, and those of urban origin together with their urban dwelling sibs were assessed by interview, examination, and fasting blood samples. Obesity, diabetes, and other cardiovascular risk factors were compared. A total of 6,510 participants (42% women) were recruited. Among urban, migrant, and rural men the age- and factory-adjusted percentages classified as obese (body mass index [BMI] >25 kg/m^2^) were 41.9% (95% confidence interval [CI] 39.1–44.7), 37.8% (95% CI 35.0–40.6), and 19.0% (95% CI 17.0–21.0), respectively, and as diabetic were 13.5% (95% CI 11.6–15.4), 14.3% (95% CI 12.2–16.4), and 6.2% (95% CI 5.0–7.4), respectively. Findings for women showed similar patterns. Rural men had lower blood pressure, lipids, and fasting blood glucose than urban and migrant men, whereas no differences were seen in women. Among migrant men, but not women, there was weak evidence for a lower prevalence of both diabetes and obesity among more recent (≤10 y) migrants.

**Conclusions:**

Migration into urban areas is associated with increases in obesity, which drive other risk factor changes. Migrants have adopted modes of life that put them at similar risk to the urban population. Gender differences in some risk factors by place of origin are unexpected and require further exploration.

*Please see later in the article for the Editors' Summary*

## Abstract 2

Shah Ebrahim and colleagues examine the distribution of obesity, diabetes, and other cardiovascular risk factors among urban migrant factory workers in India, together with their rural siblings. The investigators identify patterns of change of cardiovascular risk factors associated with urban migration.

## Editors' Summary

**Background:**

India, like the rest of the world, is experiencing an epidemic of diabetes, a chronic disease characterized by dangerous levels of sugar in the blood that cause cardiovascular and kidney disease, which lower life expectancy. The prevalence of diabetes (the proportion of the population with diabetes) has been increasing steadily in India over recent decades, particularly in urban areas. In 1984, only 5% of adults living in the towns and cities of India had diabetes, but by 2004, 15% of adults in urban areas were affected by diabetes. In rural areas of India, diabetes is less common than in urban areas but even here, the prevalence of diabetes is now 6%. Obesity—too much body fat—is a major risk factor for diabetes and, in parallel with the greater increase in diabetes in urban India compared to rural India, there has been a greater increase in obesity in urban areas than in rural areas.

**Why Was This Study Done?:**

Experts think that the increasing prevalence of obesity and diabetes in India (and in other developing countries) is caused in part by increased consumption of saturated fats and sugars and by reduced physical activity, and that these changes are related to urbanization—urban expansion into the countryside and migration from rural to urban areas. If living in an urban setting is a major determinant of obesity and diabetes risk, then people migrating into urban areas should acquire the high risk of the urban population for these two conditions. In this cross-sectional study (a study in which participants are studied at a single time point), the researchers investigate whether rural to urban migrants in India have a higher prevalence of obesity and diabetes than rural nonmigrants. They also ask whether migrants have a prevalence of obesity and diabetes intermediate between that of life-long urban and rural dwellers and whether a longer time since migration is associated with a higher prevalence of obesity and diabetes.

**What Did the Researchers Do and Find?:**

The researchers recruited rural-urban migrants working in four Indian factories in north, central, and south regions and their spouses (if they were living in the same town) into their study. Each migrant worker and spouse asked one nonmigrant brother or sister (sibling) still living in their place of origin to join the study. The researchers also enrolled nonmigrant factory workers and their urban siblings into the study. All the participants (more than 6,500 in total) answered questions about their diet and physical activity and had their fasting blood sugar and their body mass index (BMI; weight in kg divided by height in meters squared) measured; participants with a fasting blood sugar of more than 7.0 nmol/l or a BMI of more than 25 kg/m^2^ were classified as diabetic or obese, respectively. 41.9% and 37.8% of the urban and migrant men, respectively, but only 19.0% of the rural men were obese. Similarly, 13.5% and 14.3% of the urban and migrant men, respectively, but only 6.2% of the rural men had diabetes. Patterns of obesity and diabetes among the women participants were similar. Finally, although the prevalence of diabetes and obesity was lower in the most recent male migrants than in those who had moved more than 10 years previously, this difference was small and not seen in women migrants.

**What Do These Findings Mean?:**

These findings show that rural-urban migration in India is associated with rapid increases in obesity and in diabetes. They also show that the migrants have adopted modes of life (for example, reduced physical activity) that put them at a similar risk for obesity and diabetes as the urban population. The findings do not show, however, that migrants have an intermediate prevalence of obesity and diabetes compared to urban and rural dwellers and provide only weak support for the idea that a longer time since migration is associated with a higher risk of obesity and diabetes. Although the study's cross-sectional design means that the researchers could not investigate how risk factors for diabetes evolve over time, these findings suggest that urbanization is helping to drive the diabetes epidemic in India. Thus, targeting migrants and their families for health promotion activities and for treatment of risk factors for obesity and diabetes might help to slow the progress of the epidemic.

**Additional Information:**

Please access these Web sites via the online version of this summary at http://dx.doi.org/10.1371/journal.pmed.1000268.

- The International Diabetes Federation provides information about all aspects of diabetes, including information on diabetes in Southeast Asia (in English, French, and Spanish)
- DiabetesIndia.com provides information on the Indian Task Forces on diabetes care in India
- Diabetes Foundation (India) has an international collaborative research focus and provides information about health promotion for diabetes; it has also produced consensus guidelines on dietary change for prevention of diabetes in India
- The US National Diabetes Information Clearinghouse provides detailed information about diabetes for patients, health care professionals, and the general public (in English and Spanish)
- MedlinePlus provides links to further resources and advice about diabetes (in English and Spanish)

## Introduction

In India the urban prevalence of diabetes in adults has risen from 5% in 1984 to just under 15% in 2004 [1],[2]. Markedly lower rural levels of diabetes have been evident for decades, but more recently prevalence appears to have increased from 2% to 6% in rural south India [3]. Underlying these adverse trends in diabetes are increases in obesity affecting urban areas much more than rural areas of India. The 2nd Indian National Family Household Survey in 1998–1999 confirmed the marked rural-urban differences in prevalence of obesity among women [4] and men [5], and also a rising trend between the 2nd and 3rd National Family Household Survey in 2005 [6]. The increasing risks of obesity and diabetes in India and in other low- and middle-income countries have been attributed to increased consumption of saturated fats, sugars, and sedentary behaviour associated with urbanisation and westernisation [7]. However, obesity and diabetes have early life origins that track into adulthood and these may play a critical role in explaining the obesity and diabetes “epidemics” in developing countries [8]. In India urbanisation is caused by urban expansion into peripheral areas and internal migration from rural to urban areas, largely for economic reasons. However, it is not clear how urbanisation increases the risk of obesity and diabetes among people who have had divergent early life experiences, particularly in developing countries. Migration studies are powerful means of identifying environmental causes of common diseases as changes in environment are large and occur at a known time, making causal inferences more feasible. In a major review of the evidence on migration and cardiovascular risk factors and obesity, McKay and colleagues state “it is clear that migrants in general tend to suffer from worse health and display disadvantaged risk factor profiles. In comparison to the host population they are more frequently subject to hypertension, chronic conditions, low birth weight, and obesity etc. Moreover, their ill health and unfavourable risk profiles may worsen with increasing duration of stay” [9]. While the trends of increased risk of obesity and diabetes among both international south Asian migrants are well documented [10],[11], much less is known about the effects of internal rural-to-urban migration in India.

The rising rates of diabetes in both urban and rural India indicate that urbanisation is an important but not a sufficient explanation. Examining the experience of rural-to-urban migrants would help understand what is driving these trends. Migrants would be expected to acquire the high risk of the urban population if the disease is largely environmentally determined and for such trends to be dependent on the duration of time spent in a new environment and the extent to which traditional ways of living are lost—as seen for coronary heart disease among the Japanese moving to the US [12]. Conversely, for some common causes of chronic disease in the country of origin, the environmental changes associated with migration may result in lower risk of hypertensive heart disease, for example [13]. Previous studies are conflicting with some suggesting that changes in cardiovascular risk factors (particularly blood pressure) may occur within a few years [14], whilst others indicating that considerable exposure to urban life is necessary [9]. A recent review of the experience of migrants to the US indicates a complex picture with generally better health among migrants but with heterogeneity between different groups, probably reflecting the duration of stay in the US [15].

Based on these findings, the main hypothesis of our study was that rural-urban migrants would have higher rates of obesity and diabetes than rural nonmigrants and secondary hypotheses were that (a) rural-urban migrants' rates of obesity and diabetes would be intermediate to that of rural and life-long urban dwellers and (b) longer stays in the urban environment would increase rates of obesity and diabetes.

## Methods

Using the framework of a cardiovascular risk factor screening study conducted in factories in north, central, and south India [16], we designed a sib-pair comparison study. Details of the design have been reported elsewhere [17]. Briefly, the study was in four Indian factories (Lucknow, Hindustan Aeronautics Ltd; Nagpur, Indorama Synthetics Ltd; Hyderabad, Bharat Heavy Electricals Ltd; and Bangalore, Hindustan Machine Tools Ltd) situated in the north, centre, and south of the country. Factory workers and their coresident spouses were recruited if they were rural-urban migrants using employer records as the sampling frame. Each migrant worker and spouse was asked to invite one nonmigrant full sibling of the same sex and closest to them in age still residing in their rural place of origin. Precedence was given to gender over age and where multiple same-sex sibs were available the one closest in age was invited. This strategy resulted in rural dwelling sibs being drawn from 20 of the 29 states in India, reflecting the migration patterns of the factory workforce and their spouses. A 25% random sample of nonmigrants was invited to participate in the study. Nonmigrants were also asked to invite a sib who resided in the same city but did not work in the factory. Information sheets were translated into local languages and signed (or a thumb print used if the individual was illiterate), and through this, informed consent obtained. Ethics committee approval was obtained from the All India Institute of Medical Sciences Ethics Committee, reference number A-60/4/8/2004. Field work began in March 2005 and was completed by December 2007.

### Measurement of Cardiovascular Risk Factors

Standing height was measured with mandibular stretch at end expiration using a plastic stadiometer (Leicester height measure; Chasmors Ltd), and weight was measured in light clothes with shoes off using a digital scale (Model PS16). Skinfold thickness was measured three times at the triceps, subscapular, and medial calf using Holtain calipers and the average of the three measures used. Subscapular and triceps skinfolds were used to calculate percent body fat using a standard formula [18]. Waist and hip circumferences were measured using a nonstretch narrow metal tape with a blank lead in (Chasmors metallic tape), taking the average of two readings. Blood pressure was measured using an Omron M5-I automatic machine in the sitting position using the right upper arm and an appropriate sized cuff after a period of 5 min rest. Participants were interviewed using a structured questionnaire to obtain information about tobacco use and alcohol consumption.

### Obesity- and Diabetes-Related Outcomes

Obesity was defined as body mass index (BMI) greater than 25 kg/m^2^ (Indian adult population standard) [19]. A diagnosis of diabetes was made using the World Health Organization (WHO) fasting plasma glucose criterion of >7.0 mmol/l [20] or report of a doctor diagnosis of diabetes. Homeostasis model assessment (HOMA) scores to estimate insulin resistance were calculated from fasting blood glucose and serum insulin levels using a standard formula of plasma glucose (mol/l) × plasma insulin (mU/l)/22.5), on the basis of the original approach [21]. HOMA has been validated by comparison with biochemical markers of insulin resistance in healthy Indian people, yielding moderate correlations [22].

### Dietary Assessment

Diet was assessed by an interviewer-administered semiquantitative food frequency questionnaire (FFQ). The questionnaire assessed frequency of intake (daily, weekly, monthly, yearly/never) of 184 commonly consumed food items. In order to assess the reliability of the FFQ, subsamples were asked to complete the questionnaire 1–2 mo (*n* = 185), as well as 12 mo (*n* = 305) after completion of the questionnaire during the original period of data collection. Kappa coefficients ranging from 0.26–0.71 were obtained, which are similar to values obtained in other reliability studies [23],[24]. Another 530 participants (53.9% males) were administered a reference method of three 24-h recalls, which was used to validate the FFQ. Most food items yielded validities that were acceptable. Fat intake (g/d) was reliably measured and is presented here as an indicator of dietary change.

### Physical Activity

An interviewer-administered questionnaire was used to assess physical activity of the past month across multiple domains including discretionary leisure time, household chores, work, sleep, sedentary activities, and other common daily activities. For each activity the average amount of time and the frequency were documented. Participants reported frequencies to fixed categories of “daily,” “once a week,” “2–4 times a week,” “5–6 times a week,” “once a month,” and “2–3 times a month.” Metabolic equivalent tasks (METs) were estimated as the ratio of resting metabolic rate where 1 MET is equivalent to the energy expenditure value of sitting quietly. When all the activities reported did not cumulatively account for 24 h, a standard MET of 1.4 was applied to the residual time [25]. For manual occupational activity an integrated energy index (IEI) of the activity was applied instead of the absolute MET value. IEI take into account “rest” or “pause” periods, which individuals are likely to take when engaged in these manual activities [26]. Validation of the questionnaire was conducted in 49 rural and 45 urban participants by making comparisons with uni-axial accelerometers and a 24-h activity diary. Physical activity showed acceptable validity with these reference methods with little evidence of bias although correlations were only modest (accelerometer *r* = 0.28; *p*<0.01; 24-h activity diary *r* = 0.30; *p*<0.01) [27]. Activity data were summarised as METs hours per day.

### Socioeconomic Position

A subset of 14 of 29 questions were used from the Standard of Living Index (SLI), a household level asset-based scale devised for Indian surveys [5], selecting those we believed most informative for our study population. They comprised: quality of house; toilet facilities; source of lighting and drinking water; possession of clock, radio, television, bicycle, motorcycle, car, tractor, refrigerator, telephone; and weighted to give a maximum score of 38. Weights of items for the SLI were developed by the International Institute of Population Sciences in India [5], and were based on a priori knowledge about the relative significance of the items. These same weights were used for the Indian Migrants Study analysis. Measurement at the household level is appropriate in the Indian context, in which the individual's socioeconomic position has less impact on their material wealth. This asset-based score was considered a more appropriate indicator of socioeconomic position for these analyses than education, income, or occupation because it is more likely to reflect the changes that migrants experience following their move to urban areas. A low SLI is associated with tobacco use [28] and with mortality [29], indicating its validity as a socioeconomic marker.

### Laboratory Assays

Participants were asked to attend fasting and the time of last meal was recorded. Blood samples, with the exception of glucose assays, were separated and stored at −20°C locally and transported monthly to the All India Institute of Medical Sciences (AIIMS), Delhi. Glucose was measured on the day of sample collection in local laboratories at each site with the GOD-PAP method using RANDOX kits [30]. Serum HDL cholesterol was estimated directly by an elimination method [31], total cholesterol was estimated by an enzymatic endpoint method, and triglycerides by GPO-PAP method. The quality of local assays was checked with regular external standards and internal duplicate assays and monitored by AIIMS. For quality assurance the Cardiac Biochemistry Lab, AIIMS, is part of the UK National External Quality Assessment (http://www.ukneqas.org.uk/).

### Statistical Analyses

As the study is based on factory workers, their spouses, and a sibling of each factory worker and spouse these data cannot be treated as coming from independent individuals and the data structure must be accounted for in the statistical analysis. The focus of the present analyses was the comparison between the three groups: urban, migrants, and rural. Of these, the migrant and rural groups were paired with each other (with a sibling in each group), while the urban group of siblings was an independent referent not paired to the other two groups. A general model framework that can accommodate this data structure was therefore required, leading us to use multilevel (i.e., random effects) models [32]. In the multilevel model the between-pair variation is specified explicitly and included in the model. The models include a random shift in the intercept applied to both siblings from a pair. This makes two siblings similar (within-pair correlation) and at the same time different to individuals from other sib pairs (between-pair variation). Comparisons were made between nonmigrant urban dwellers, rural-urban migrants, and nonmigrant rural dwellers using linear random effects models for the continuous outcomes and for the binary outcomes logistic regression with a pair-specific random effect to estimate the within-pair comparisons.

Men and women were analysed separately as we anticipated that there might be gender differences in migration effects. Separate analyses of men and women were also more readily interpreted given the statistical dependency between husbands and wives produced by the study design. Adjustments were made for factory and the interaction between age and age group in all comparisons. As the urban group was expected to have the highest and the rural group the lowest levels of risk factors and disease, trend tests were carried out scoring the groups 1 to 3 and using likelihood ratio tests. As a secondary hypothesis, tests of whether the effects in the urban and migrant groups were equal were also made. All analyses were conducted using STATA 10.

## Results

### Response Rates

Employee records indicated that 21,662 workers and spouses at the four factories were available for study. A total of 15,596 (72%) of these individuals were identified as still working in the factory and were contacted, of whom 13,695 (88%) completed an assessment of their eligibility for the study. Of those who completed this initial assessment, 7,594 (55%) were eligible for inclusion as they had a rural dwelling sib or were selected as part of a random 25% sample of urban nonmigrants. Of eligible individuals, 7,102 (94%) agreed in principle to complete the clinical examination with their sibling, of whom 3,537 (50%) sib-pairs eventually participated by the close of field work. Factory workers who lived in rural areas and commuted to work (*n* = 519) and 38 urban-rural migrants were excluded from these analyses (see Figure S1). Failure to participate was largely due to unwillingness of rural sibs to travel and competing time pressures (school exams, harvest season). Limited data were available from the initial screening interview to make comparisons between responders, nonresponders, and those unwilling to consent in the full clinical study. No differences in marital status, mean age, distance from rural place of origin, and migrant status were found (see Table S1). Self-reported prevalence of cardiovascular diseases was lower in nonresponders (14.8%), but higher in nonconsenters (21.1%), than in responders (19.3%). There were differences in smoking habits between responders and other groups.

### Demographic and Socioeconomic Characteristics

A total of 6,510 participants were included in analyses of whom 2,723 (42%) were women; overall 2,287 were nonmigrant urban dwellers, 2,112 rural-urban migrants, and 2,111 nonmigrant rural dwellers (see Table 1). Urban men were older than rural men but of similar age to migrant men. Education to at least primary school level was near universal among urban men and women, and migrant men, but was less likely for rural women. The median standard of living index was similar for both urban and migrant households but was considerably lower for rural ones. Most migrants had spent a considerable time in the urban environment (median, range: men 26 y [1–55 y] and women 21 y [<1–49 y]) with 9.3% (95% confidence interval [CI] 7.6–11.0) of men and 21.3% (95% CI 18.7–23.9) of women spending ≤10 y (see Figure S2).

**Table 1:** Study population characteristics by sex and place of origin, Indian migration study, 2005–2007.

Study Population Characteristics	Men					Women				
	Urban	Migrant	Rural	Total	*p*-Value	Urban	Migrant	Rural	Total	*p*-Value
***n***	1,201	1,127	1,459	3,787	—	1,086	985	652	2,723	—
**Age (y), mean (SD) (min–max)**	41.5 (10.0) (18–70)	44.7 (8.6) (25–65)	39.6 (11.6) (17–76)	41.8 (10.5) (17–76)	<0.0001	39.7 (9.6) (18–68)	39.6 (8.7) (19–59)	41.4 (11.4) (17–70)	40.1 (9.8) (17–70)	0.0004
**Percent married (** ***n*** **)**	84.5 (1,015)	98.3 (1,108)	81.2 (1,184)	87.3 (3,307)	<0.0001	87.8 (953)	97.9 (964)	75.9 (495)	88.6 (2,412)	<0.0001
**Percent nonmanual worker (** ***n*** **)**	47.5 (570)	38.6 (435)	21.6 (315)	34.9 (1,320)	<0.0001	17.1 (186)	5.4 (53)	7.7 (50)	10.6 (289)	<0.0001
**Percent primary education + (** ***n*** **)**	98.8 (1,187)	98.9 (1,115)	88.1 (1,285)	94.7 (3,587)	<0.0001	96.7 (1,050)	79.9 (787)	65.0 (424)	83.0 (2,261)	<0.0001
**Standard of living index, mean (SD) (min–max)**	24.5 (5.0) (6–38)	25.1 (4.3) (11–36)	17.3 (6.7) (2–38)	21.9 (6.6) (2–38)	<0.0001	24.7 (5.2) (4–38)	24.7 (4.2) (11–34)	16.3 (6.8) (2–34)	22.7 (6.4) (2–38)	<0.0001

*p*-Likelihood ratio test for no difference between the three groups for age and standard of living index ignoring the correlated data. For the remaining factors *p*-value is the result of a simple χ^2^ test for independence.

SD, standard deviation.

### Comparisons between Urban, Migrant, and Rural Groups

**Figure 1:**
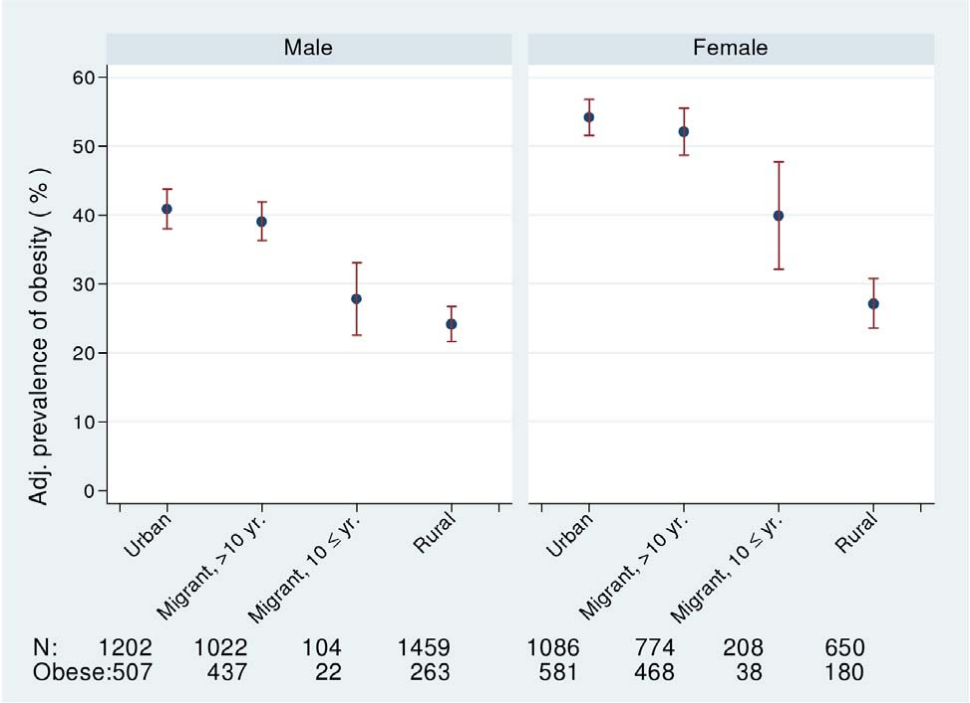
Age-, factory-, and occupation-adjusted percent prevalence (95% CI) of obesity, BMI >25 kg/m2, by migrant group and sex, Indian migration study 2005–2007. Including number of participants with information about obesity and number of obese.

Comparisons of risk factors and health problems between urban, migrant, and rural groups are shown in Tables 2 and 3 with a random effect to take account of the sib-pair design. There was strong evidence in both men and women of differences in BMI (weight/height^2^) between urban, migrant, and rural people (*p*_trend_ <0.0001) as shown in Table 2. Obesity prevalence (BMI >25 kg/m^2^) was greatest in urban women (53.5%, 95% CI 50.5–56.5) and lowest in rural men (18.0%, 95% CI 17.0–21.0), with migrants in an intermediate position (see Figure 1). The age, occupation, and factory adjusted odds of obesity were between 3- and 4-fold greater in migrant than rural men and women (Table 3). Percentage body fat estimated from skinfold thicknesses showed markedly higher values among women than men, with similar levels among urban and migrant groups, but lower levels among rural dwellers.

**Table 2:** Risk factors for cardiovascular disease.

Risk Factors	Men					Women				
	Urban	Migrants	Rural	*p* for Trend	Test Urban = Migrant	Urban	Migrants	Rural	*p* for Trend	Test Urban = Migrant
**BMI (kg/m^2^)**	24.3 (24.1–24.5)	24.0 (23.8–24.2)	21.9 (21.7–22.1)	<0.0001	0.15	25.9 (25.6–26.1)	25.2 (24.9–25.4)	22.5 (22.2–22.8)	<0.0001	0.0007
**Height (cm)**	165.7 (165.3–166.1)	165.9 (165.6–166.3)	165.6 (165.3–166.0)	0.47	0.43	153.3 (152.9–153.7)	152.8 (152.5–153.2)	152.4 (152.0–152.8)	0.002	0.06
**Waist∶hip ratio**	0.92 (0.92–0.92)	0.93 (0.92–0.93)	0.90 (0.90–0.90)	<0.0001	0.02	0.81 (0.81–0.82)	0.82 (0.82–0.83)	0.81 (0.80–0.81)	0.18	0.0006
**Percent body fat**	25.2 (24.9–25.5)	25.8 (25.5–26.1)	21.7 (21.4–22.0)	<0.0001	0.01	32.2 (31.9–32.6)	32.1 (31.8–32.5)	28.8 (28.4–29.3)	<0.0001	0.75
**SBP (mmHg)**	125.7 (124.8–126.7)	125.1 (124.2–126.1)	122.9 (122.1–123.7)	<0.0001	0.38	119.4 (118.4–120.4)	118.9 (117.9–119.9)	118.9 (117.7–120.2)	0.80	0.53
**Total cholesterol (mmol/l)**	4.74 (4.67–4.81)	4.76 (4.69–4.82)	4.56 (4.50–4.62)	<0.0001	0.77	4.81 (4.73–4.88)	4.79 (4.72–4.87)	4.74 (4.65–4.82)	0.23	0.82
**LDL cholesterol (mmol/l)**	2.89 (2.83–2.95)	2.92 (2.86–2.97)	2.76 (2.71–2.81)	0.0003	0.51	2.96 (2.89–3.02)	2.97 (2.91–3.03)	2.89 (2.82–2.97)	0.21	0.83
**Triglycerides** a **(mmol/l)**	1.41 (1.38–1.45)	1.39 (1.35–1.43)	1.29 (1.26–1.32)	<0.0001	0.41	1.32 (1.29–1.36)	1.25 (1.21–1.28)	1.26 (1.22–1.31)	0.04	0.004
**Fasting blood glucose** a **(mmol/l)**	5.30 (5.24–5.36)	5.28 (5.22–5.34)	5.12 (5.07–5.18)	<0.0001	0.64	5.19 (5.12–5.25)	5.13 (5.07–5.19)	5.09 (5.02–5.16)	0.05	0.18
**Fasting insulin** a **(mU/l)**	6.01 (5.71–6.34)	5.73 (5.44–6.04)	4.66 (4.45–4.88)	<0.0001	0.21	5.68 (5.36–6.00)	5.87 (5.56–6.20)	5.34 (4.99–5.71)	0.19	0.40
**HOMA score** a	1.31 (1.24–1.39)	1.27 (1.20–1.34)	1.02 (0.97–1.07)	<0.0001	0.43	1.24 (1.17–1.32)	1.27 (1.20–1.35)	1.17 (1.09–1.26)	0.25	0.53
**MET h/day**	37.89 (37.64–38.13)	37.93 (37.68–38.19)	39.94 (39.72–40.16)	<0.0001	0.79	36.78 (36.57–36.98)	36.82 (36.62–37.03)	37.87 (37.61–38.13)	<0.0001	0.75
**Fat intake** a **(g/day)**	90.86 (88.60–93.17)	88.00 (85.92–90.13)	71.97 (70.46–73.51)	<0.0001	0.07	75.83 (73.90–77.82)	71.17 (69.41–72.97)	58.93 (57.14–60.77)	<0.0001	0.0006

Adjusted mean (95% CI) by place of origin for men and women, adjusted for occupation, age, age group, and factory including a random effect of sibling pair.

a Geometric mean.

LDL, low-density lipoprotein; SBP, systolic blood pressure.

**Table 3 pmed-1000268-t003:** Odds ratios (95% CI) for the risk of disease in a sibling compared to a rural sibling, adjusted for occupation, age, age group, and factory with an individual-specific random effect of sib-pair.

Risk Factors	Men					Women				
	Urban	Migrants	Rural	*p* for Trend^a^	Test Urban = Migrant	Urban	Migrants	Rural	*p* for Trend^a^	Test Urban = Migrant
**Hypertension**	1.76 (1.37–2.27)	1.67 (1.31–2.12)	1	<0.0001	0.67	1.55 (1.13–2.12)	1.22 (0.90–1.65)	1	0.005	0.08
**Obese**	3.83 (2.95–4.98)	3.12 (2.44–3.98)	1	<0.0001	0.08	4.89 (3.56–6.72)	3.86 (2.88–5.19)	1	<0.0001	0.05
**Underweight**	0.21 (0.14–0.31)	0.10 (0.06–0.16)	1	<0.0001	0.002	0.23 (0.14–0.38)	0.18 (0.11–0.31)	1	<0.0001	0.37
**Diabetic**	2.43 (1.72–3.43)	2.15 (1.55–3.00)	1	<0.0001	0.42	2.96 (1.69–5.17)	2.68 (1.59–4.52)	1	0.0001	0.64
**Fasting blood glucose >7 mmol/l**	2.33 (1.46–3.73)	2.38 (1.51–3.76)	1	0.0006	0.92	2.38 (1.18–4.80)	2.26 (1.13–4.51)	1	0.02	0.83
**Regular alcohol**	1.42 (1.08–1.88)	1.38 (1.05–1.73)	1	0.007	0.70	0.31 (0.11–0.86)	0.63 (0.28–1.42)	1	0.02	0.15
**Current smoker**	0.82 (0.66–1.03)	0.61 (0.49–0.75)	1	0.03	0.01	0.28 (0.09–0.89)	0.66 (0.27–1.63)	1	0.02	0.11
**Physically inactive**	2.00 (1.66–2.41)	1.62 (1.33–1.97)	1	<0.0001	0.02	1.14 (0.87–1.50)	1.20 (0.92–1.57)	1	0.41	0.65

a Test for trend on the log-odds scale.

The urban and migrant groups were both very similar regarding MET h/d of physical activity, whereas the rural group had a higher average MET h/d (Table 2). This pattern was still seen after adjusting for BMI (Table 4). Participants were considered to be physically inactive if they belonged to the lowest third of MET h/d (for men and women separately). There was a significant trend over the three groups for men with the highest odds of being physically inactive in the urban group (see Table 3). There was no clear pattern for women. For both men and women the urban group had the highest levels of fat intake followed by the migrants and the rural group had the lowest level (Tables 2 and 4).

**Table 4:** Adjusted mean (95% CI) by place of origin for men and women, adjusted for BMI, occupation, age, age group, and factory including a random effect of sibling pair.

Risk Factors	Men					Women				
	Urban	Migrants	Rural	*p* for Trend	Test Urban = Migrant	Urban	Migrants	Rural	*p* for Trend	Test Urban = Migrant
**SBP (mmHg)**	124.9 (124.0–125.9)	124.3 (123.4–125.2)	124.2 (123.4–125.0)	0.51	0.34	118.8 (117.8–119.9)	118.6 (117.6–119.6)	120.5 (119.2–121.8)	0.05	0.74
**Total cholesterol (mmol/l)**	4.70 (4.63–4.77)	4.71 (4.65–4.78)	4.63 (4.57–4.69)	0.08	0.86	4.79 (4.71–4.86)	4.78 (4.71–4.85)	4.79 (4.70–4.88)	0.96	0.90
**LDL cholesterol (mmol/l)**	2.86 (2.80–2.92)	2.89 (2.83–2.94)	2.80 (2.75–2.86)	0.11	0.60	2.94 (2.88–3.01)	2.96 (2.90–3.02)	2.93 (2.85–3.01)	0.87	0.76
**Triglycerides** a **(mmol/l)**	1.38 (1.34–1.42)	1.36 (1.32–1.39)	1.34 (1.31–1.38)	0.15	0.35	1.31 (1.27–1.34)	1.24 (1.20–1.27)	1.31 (1.27–1.36)	0.99	0.01
**Fasting blood glucose** a **(mmol/l)**	5.27 (5.21–5.34)	5.26 (5.20–5.32)	5.16 (5.11–5.21)	0.005	0.69	5.17 (5.11–5.24)	5.12 (5.06–5.18)	5.12 (5.04–5.20)	0.28	0.20
**Fasting insulin** a **(mU/l)**	5.60 (5.32–5.89)	5.34 (5.08–5.61)	5.23 (5.00–5.47)	0.05	0.19	5.46 (5.16–5.78)	5.74 (5.44–6.05)	5.89 (5.50–6.30)	0.10	0.21
**HOMA score** a	1.23 (1.17–1.30)	1.19 (1.13–1.25)	1.15 (1.09–1.20)	0.06	0.34	1.20 (1.13–1.27)	1.25 (1.18–1.32)	1.30 (1.21–1.39)	0.10	0.33
**MET h/d**	37.94 (37.69–38.19)	38.00 (37.74–38.26)	39.84 (39.61–40.07)	<0.0001	0.75	36.81 (36.60–37.01)	36.85 (36.64–37.06)	37.77 (37.51–38.04)	<0.0001	0.75
**Fat intake** a **(g/d)**	89.64 (87.42–91.91)	86.70 (84.64–88.80)	73.67 (72.09–75.29)	<0.0001	0.06	75.36 (73.43–77.34)	70.76 (69.02–72.55)	60.22 (58.35–62.16)	<0.0001	0.0006
**Hypertension** b	1.25 (0.96–1.63)	1.19 (0.93–1.53)	1	0.10	0.71	0.94 (0.67–1.31)	0.78 (0.56–1.07)	1	0.92	0.18
**Diabetic** b	1.86 (1.31–2.65)	1.65 (1.17–2.32)	1	0.0007	0.43	1.85 (1.06–3.24)	1.79 (1.05–3.03)	1	0.05	0.86
**Fasting blood glucose >7 mmol/l** b	1.78 (1.10–2.88)	1.86 (1.16–2.98)	1	0.03	0.82	1.62 (0.80–3.31)	1.61 (0.80–3.25)	1	0.25	0.97

a Geometric mean.

b Odds ratios (95% CI) for the risk of disease in a sibling compared to a rural sibling, adjusted for BMI, occupation, age, age group, and factory with an individual-specific random effect of sib-pair.

LDL, low-density lipoprotein; SBP, systolic blood pressure.

Smoking and drinking alcohol were rare among women; among men, migrants reported the lowest prevalence of smoking and rural men the highest. Alcohol use was highest among migrant men and lowest in rural men. Odds of hypertension (i.e., doctor diagnosis, on blood pressure lowering drugs, or blood pressure >140/90) in urban and migrant men were almost twice those of rural men and evidence of increased odds were also seen in women. Blood cholesterol and triglycerides were similar in urban and migrant groups but values were lower in rural men (*p*_trend_ <0.0001), with no differences observed in these variables in women.

**Figure 2:**
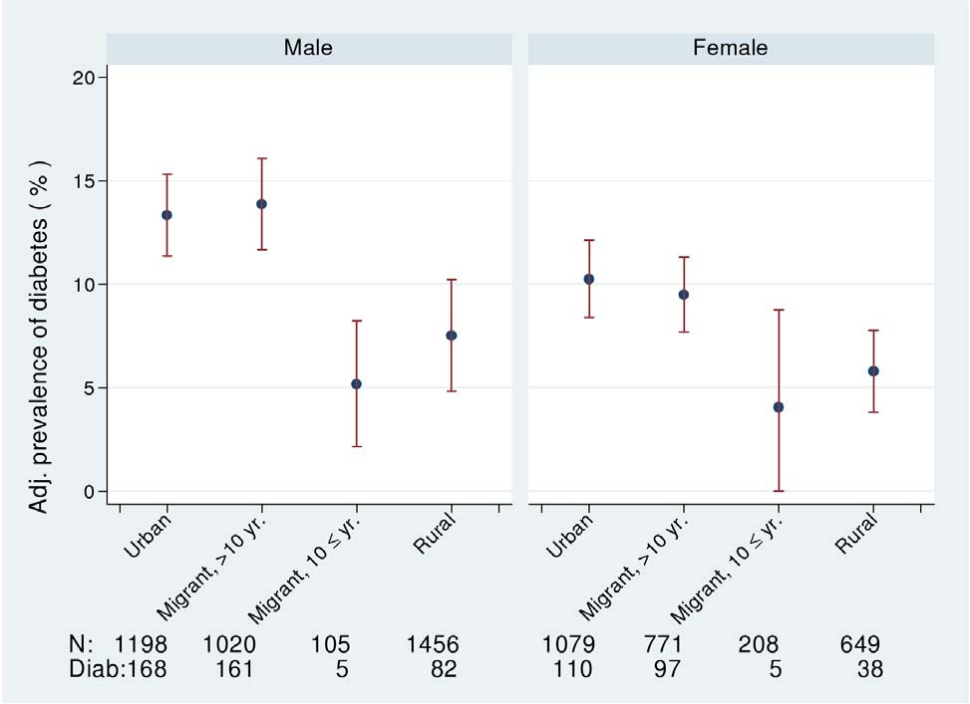
Age-, factory-, and occupation-adjusted percent prevalence (95% CI) of diabetes (diagnosed, on treatment, or fasting glucose >7 mmol/l) by type of migrant and sex, Indian migration study 2005–2007. Including number of participants with information about diabetes and number of diabetics.

In both men and women, fasting blood glucose levels were similar in urban and migrant groups and lowest in rural groups (*p*_trend_ <0.0001). In men, but not women, fasting insulin levels and HOMA scores showed a downward trends (*p*_trend_ ≤0.0001) from urban, migrant, to rural. The prevalence of diabetes (i.e., doctor diagnosis, on treatment, or fasting blood glucose >7.0 mmol/l) was higher in urban and migrant groups than the rural group (see Figure 2). Both urban and migrant men and women had over 2-fold increased odds of diabetes compared with rural participants.

Further adjustment for BMI in men weakened the associations between place of origin and systolic blood pressure, hypertension, total cholesterol, and triglycerides but did not reduce the strength of associations with fasting blood glucose, HOMA, or the prevalence of diabetes (see Table 4). In women, further adjustment for BMI weakened the associations between place of origin and hypertension. Adjustment for percent body fat computed from skinfold thicknesses instead of BMI produced similar effects on associations (unpublished data).

### Between Sib-Pair Differences

Data in Table 5 correspond to Tables 2–4 but focus on the estimated contrasts between migrant and rural sibs. The values shown represent the average difference between the migrant and nonmigrant rural sib. For example, the BMI of the male migrant sib group was 2.10 kg/m^2^ (95% CI 1.84–2.37 kg/m^2^) greater than that of the rural sib group of the same age. Among men the migrant sib group had consistently more adverse measures of obesity, lipids, and diabetes than the rural sib group. The between sib group differences were modest, 4.08% (95% CI 3.7–4.47) difference in body fat, 2.2 mmHg (95% CI 1.0–3.4 mmHg) in systolic blood pressure, and 0.20 mmol/l (95% CI 0.11–0.28 mmol/l) in total cholesterol. Comparing the migrant with the rural male sibs, HOMA scores were 1.25- (95% CI 1.16–1.34) fold higher, triglycerides were 1.08- (95% CI 1.04–1.12) fold higher, and fasting glucose was 1.03- (95% CI 1.02–1.05) fold higher. Adjustment for BMI attenuated these small differences between migrant and urban sibs. Differences were less marked between migrant and rural women.

**Table 5:** Estimated contrast (95% CI) between migrant and rural sibling for men and women adjusted for age, age group, and factory including a random effect of sibling pair.

Risk Factors	Men		Women	
	Adjusting For Age Group, Occupation, and Factory	Adjusting For Age Group, Occupation, Factory, and BMI	Adjusting For Age Group, Occupation, and Factory	Adjusting For Age Group, Occupation, Factory, and BMI
**BMI (kg/m^2^)**	2.10 (1.84–2.37)	—	2.65 (2.25–3.06)	—
**Standing height (cm)**	0.32 (−0.12 to 0.77)	—	0.41 (−0.10 to 0.92)	—
**Waist∶hip ratio**	0.03 (0.02–0.03)	—	0.01 (0.01–0.02)	—
**Percent body fat**	4.08 (3.70–4.47)	—	3.29 (2.82–3.76)	—
**SBP (mmHg)**	2.21 (1.02–3.40)	0.08 (−1.14 to 1.30)	−0.02 (−1.55 to 1.52)	−1.90 (−3.47 to −0.33)
**Total cholesterol (mmol/l)**	0.20 (0.11–0.28)	0.08 (−0.00 to 0.17)	0.06 (−0.05 to 0.16)	−0.01 (−0.12 to 0.10)
**LDL cholesterol (mmol/l)**	0.16 (0.08–0.23)	0.08 (0.01–0.16)	0.08 (−0.02 to 0.17)	0.02 (−0.07 to 0.12)
**Triglycerides** a **(mmol/l)**	1.08 (1.04–1.12)	1.01 (0.97–1.05)	0.99 (0.95–1.03)	0.94 (0.91–0.98)
**Fasting blood glucose** a **(mmol/l)**	1.03 (1.02–1.05)	1.02 (1.00–1.03)	1.01 (0.99–1.03)	1.00 (0.98–1.02)
**Fasting insulin** a **(mU/l)**	1.23 (1.15–1.31)	1.02 (0.96–1.09)	1.10 (1.01–1.19)	0.97 (0.90–1.06)
**HOMA score** a	1.25 (1.16–1.34)	1.03 (0.96–1.11)	1.09 (1.00–1.18)	0.96 (0.88–1.05)
**MET h/day**	−2.01 (−2.35 to −1.67)	−1.84 (−2.19 to −1.49)	−1.04 (−1.37 to −0.71)	−0.92 (−1.26 to −0.58)
**Fat intake** a **(g/day)**	1.22 (1.19–1.26)	1.18 (1.14–1.21)	1.21 (1.16–1.25)	1.17 (1.13–1.22)

In column 2 and 4 also adjusting for BMI. No adjustments made for variables related to BMI.

a Relative difference.

LDL, low-density lipoprotein; SBP, systolic blood pressure.

The prevalence of obesity and of diabetes was examined by stratifying years since migration (>10 y versus ≤10 y). In men, but not women, there was weak evidence for linear trends in both obesity and diabetes from rural, more recent migrants, longer-term migrants, and urban dwellers (see Figures 1 and 2; Table 6). However, there was no strong statistical evidence of differences in odds of obesity or diabetes between the two migrant groups in either men or women.

**Table 6:** Odds ratios (95% CI) for the risk of disease in a sibling compared to a rural sibling, adjusted for occupation age, age group, and factory with an individual-specific random effect of sib-pair.

Condition	Men					Women				
	Urban	Migrants >10 y	Migrants ≤10 y	Rural	*p* for Trend^a^	Urban	Migrants >10 y	Migrants ≤10 y	Rural	*p* for Trend^a^
**Obese**	3.85 (2.96–5.01)	3.24 (2.52–4.17)	2.04 (1.08–3.87)	1	<0.0001	4.90 (3.57–6.73)	3.82 (2.83–5.17)	4.53 (2.53–8.12)	1	<0.0001
**Diabetes**	2.43 (1.72–3.43)	2.17 (1.56–3.03)	1.80 (0.63–5.16)	1	<0.0001	2.97 (1.70–5.22)	2.63 (1.55–4.46)	4.70 (1.22–18.19)	1	<0.0001

The migrant group is now split in more or less than 10 y since migration.

a Test for trend on the log-odds scale.

## Discussion

Our main hypothesis that rural-urban migrants have higher prevalence rates of obesity and diabetes than rural nonmigrants was strongly supported by our findings. However, our secondary hypothesis that migrants would have intermediate prevalence compared with urban dwellers was generally not supported. Our final hypothesis, that longer time since migration would be associated with increased risk was also not supported. Migration was associated with both an increased fat intake and reduced physical activity in both men and women, as compared with rural dwellers, and this likely contributed to the higher levels of obesity and diabetes observed in migrants. The major sex differences seen in our analyses were unexpected, with migration-associated differences in blood pressure, lipids, fasting blood glucose, and insulin only seen in men.

Adjustment for BMI in our analyses resulted in attenuation of the place-of-origin effect in men for blood pressure and lipids indicating that increases in these risk factors among migrants may be mediated by obesity. It is possible that in men more of the caloric intake comprises alcohol resulting in the observed sex differences in blood pressure and lipids. Further analyses comparing married couples who both migrated at the same time might be informative in understanding sex differences in response to migration as they would share duration of migration and certain lifestyle characteristics. Furthermore, women migrating within 10 y tended to be more obese, which may indicate selection into marriage by body size as traditionally women move from rural places of origin to join their husbands in urban areas.

Most previous migrant studies have compared the experiences of migrants with the host population but have not been able to make comparisons with the places from which migrants have come. Consequently these studies have not been able to dissect out whether differences observed are due to selection effects (e.g., a healthy migrant effect) or due to maintenance of traditional healthier life styles. Studies have also indicated that earlier age at exposure to migration may increase cardiovascular risk [33]–[37]. However, migration studies are not generally able to separate out the effects of age at migration from duration of residence in the host population [38]. Among Mexican Americans it has been documented that first generation immigrants have better health despite lower socioeconomic position than white Americans [39],[40], but this relative advantage declines with length of residency in the US [41],[42]. These findings suggest that there may be considerable latency of any effect of migration on health behaviours and outcomes. In our data there is some evidence that the impact of migration on obesity and diabetes is more rapid, occurring in the first decade of migration, which confirms findings from migrants to the US [43]. However, given the relatively small numbers of migrants in our study who had been in urban places for short durations, these findings should be viewed with caution and require replication. The effects of better access to health care (provided for factory workers and their coresident families) may also influence the propensity for diagnoses of diabetes and hypertension, which will be explored in future analyses.

Interpretation of migration studies is not straightforward as differences in health outcomes may reflect influences of place of origin, exposure to new environmental factors, effects of the process of migration itself, and also selection of those who migrate [13],[44]. Moreover, migration as an “exposure” is complex, involving a wide range of socioeconomic, behavioural, and environmental changes. Here we have used counterfactual reasoning that the rural nonmigrant sib provides an adequate control for the migrant sib, thereby dissecting out the effect of migration from the general secular drift in environmental exposures and changes in health behaviours affecting both urban and rural populations. Although the data were collected using a sib-pair design, the focus of the present analysis is the comparison between the three groups of interest: urban, migrants, and rural. Of these, only the migrant and rural groups are paired with each other (with a sibling in each group), while the urban group of siblings is an independent referent not paired to the other two groups. Future analyses will focus on the sib-pair differences in more detail as there is some indication that the migrant sibs tended to be taller than the nonmigrant rural sib (Table 5), which may indicate a selection effect. The sib-comparison design has been used previously to study migration effects on cardiovascular disease in a study of Irish migration to the US in the 1950s [45]. No differences in cardiovascular risk were found between the US residents, the immigrant Irish, or Irish living in Eire. These negative findings may have been an effect of rising cardiovascular risk in Ireland and falling risk in the US [46].

The most recent national data (2005) for India gave an adult prevalence for obesity (BMI >25 kg/m^2^, Indian standard) among employed people of 20% in urban areas and 6% in rural areas [5], which is markedly lower than our prevalence of over 50% and 20% in urban and rural areas, respectively. In a large survey of six cities, an age-adjusted diabetes prevalence of 12% was reported in 2000 [47], which is lower than our urban prevalence estimate of around 15%. A recent study in urban India reported a 15% prevalence of diabetes, comparable with our estimate [48]. In comparisons with the 3rd National Family Household Survey [5] and the 2001 Census [49], our study population had lower proportions of illiterate individuals and higher proportions of individuals with access to household facilities and assets, indicating a generally wealthier and more educated population than the national average in both rural and urban areas. This finding was expected given that our sample was drawn from employed people and their relatives. Our participants reflect those in the vanguard of social and epidemiologic change.

Our findings confirm a previous report of higher levels of serum insulin in urban as compared to the rural participants [50]. This suggests that some of the effects of urbanisation may be mediated through biological factors that result in increased secretion of insulin due to tissue resistance to its actions. Our findings are consistent with other studies of migrants where high levels of serum insulin have been reported in Asian Indians living abroad [51], in populations from other developing countries experiencing rapid urbanisation, and in migrant populations elsewhere [52].

Our response rates were lower than anticipated largely because of the logistic complexity of the sib-pair design. In a majority of cases these logistics involved at least a day to travel to the study centre and a day to travel back for the rural sib; in extreme cases up to 3 d travelling each way was involved. The differences in smoking prevalence between responders and nonresponders and nonconsenters were consistent with the play of chance. The prevalence of cardiovascular disease in the nonresponders was lower than in the responders and the nonconsenters. However, when considering those who took part with those who did not take part, no strong evidence of difference is apparent. While the response rate was suboptimal, from the data we have, there does not appear to be any major bias in health status or health behaviour. Response bias would influence our findings if there was differential nonresponse by health status and place of origin. Responders did report more cardiovascular disease than nonresponders but there was no difference in place of origin. This finding would be unlikely to alter the substantial differences we observed in prevalence of obesity and diabetes in the urban compared with rural samples. A further limitation is the cross-sectional design that does not permit longitudinal measurement to examine how cardiovascular risk and diabetes evolve over time in relation to migration. It is sometimes feasible to recruit participants into migration studies prior to migration (e.g., Luo [34], Tokelu islanders [35], Yi [36] studies), which have generally demonstrated that changes in risk factors are not explained by selection effects [14]. The forced migration of large populations living in the Three Gorges dam project in China is providing an opportunity to evaluate the effects of migration longitudinally on whole populations without any selection of who migrates [53],[54], but the process has been carefully planned and will not necessarily be generalisable to the effects of more typical migration experiences. If migration effects on health outcomes in India are as rapid as appears to be the case, establishing prospective studies in areas with high rural outflow to cities would be feasible.

Migrants (particularly in the workplace) and their families are a readily identifiable group who might be more motivated to take part in health promotion activities and treatment of risk factors than the general population. The scale of obesity and diabetes among these factory workers, their spouses, and rural sibs is very large, arguing for much wider adoption of population prevention activities as proposed by the WHO [55].

## Supporting Information

Figure S1 Flow chart for participation in Indian migration study 2005–2007. *, Rural nonmigrants excluded for these analyses as they were factory workers living in rural areas and commuting to urban factory site.
(0.31 MB TIF)

Figure S2 Distribution of years spent in urban setting by migrant migrants by sex, Indian Migration Study 2005–2007.
(0.32 MB TIF)

Table S1 Participant (factory worker or spouse) characteristics by responder status.
(0.02 MB RTF)

## Abbreviations

BMI: body mass index
CI: confidence interval
HOMA: homeostasis model assessment
MET: metabolic equivalent task
