# Correction: The Effect of Rural-to-Urban Migration on Obesity and Diabetes in India: A Cross-Sectional Study

**DOI:** 10.1371/annotation/b1ecad56-652a-4a30-9920-26679d5a584a

**Published:** 2011-05-11

**Authors:** Shah Ebrahim, Sanjay Kinra, Liza Bowen, Elizabeth Andersen, Yoav Ben-Shlomo, Tanica Lyngdoh, Lakshmy Ramakrishnan, R. C. Ahuja, Prashant Joshi, S. Mohan Das, Murali Mohan, George Davey Smith, Dorairaj Prabhakaran, K. Srinath Reddy, for the Indian Migration Study group

The Academic Editor's name and institution were erroneously omitted from the metadata of the PDF and HTML versions of this article.

The Academic Editor providing expert input on this paper was Peter Byass, Umeå Centre for Global Health Research, Department of Public Health and Clinical Medicine, Umeå University, Sweden.

For more information about the role of the Academic Editor in PLoS Medicine's editorial process, see our Author Guidelines: http://www.plosmedicine.org/static/guidelines.action#overview.

