# Supplementary figures and images for: The Effect of Rural-to-Urban Migration on Obesity and Diabetes in India: A Cross-Sectional Study
Source: PLoS Med. 2010 Apr 27;7(4):e1000268. doi: 10.1371/journal.pmed.1000268 (PMC2860494; doi:10.1371/journal.pmed.1000268)

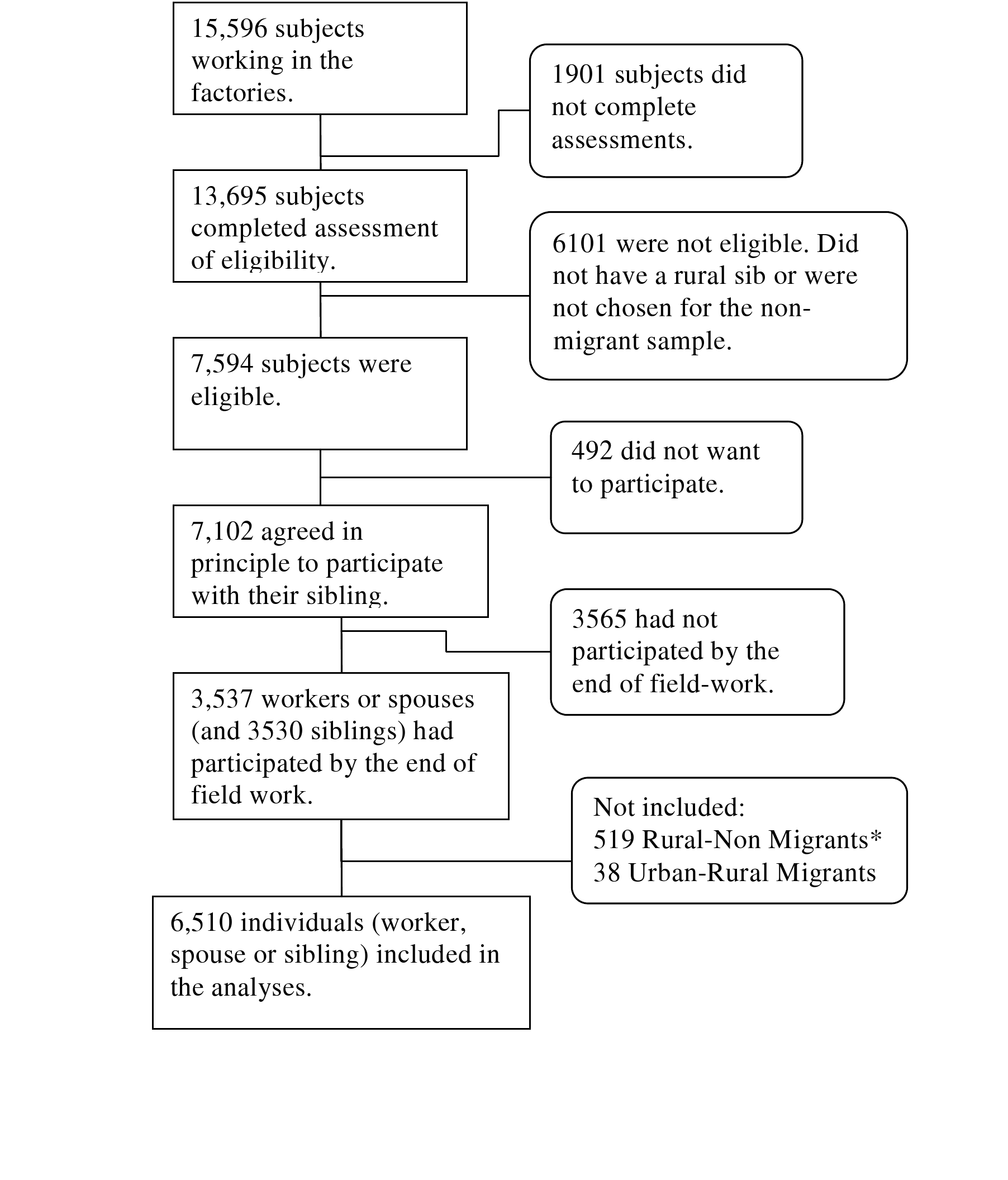

Supplement: Figure S1 — Flow chart for participation in Indian migration study 2005–2007. *, Rural nonmigrants excluded for these analyses as they were factory workers living in rural areas and commuting to urban factory site. (0.31 MB TIF) [file pmed.1000268.s001.tif]

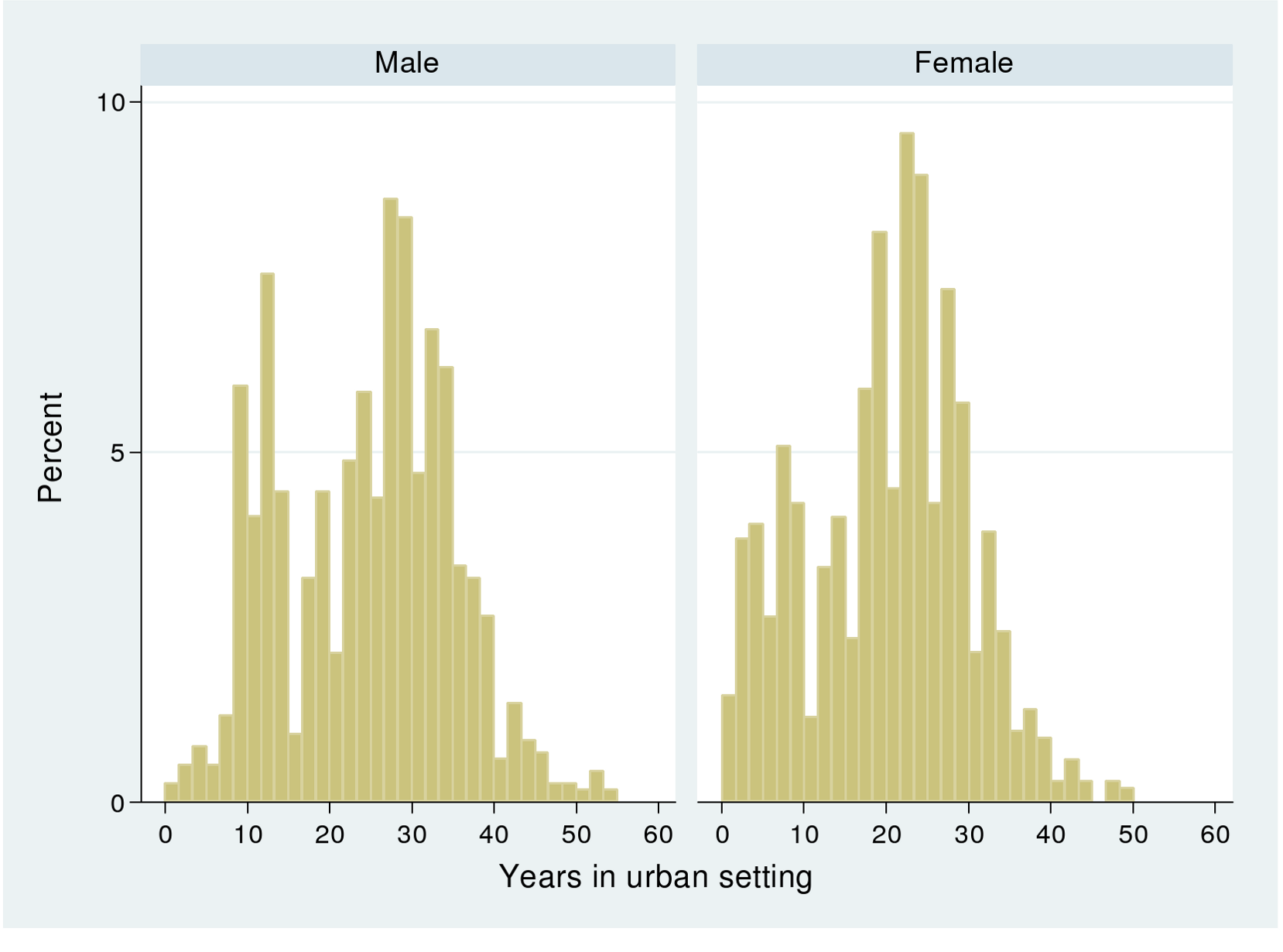

Supplement: Figure S2 — Distribution of years spent in urban setting by migrant migrants by sex, Indian Migration Study 2005–2007. (0.32 MB TIF) [file pmed.1000268.s002.tif]
