# Supplementary material for: The Effect of Rural-to-Urban Migration on Obesity and Diabetes in India: A Cross-Sectional Study
Source: PLoS Med. 2010 Apr 27;7(4):e1000268. doi: 10.1371/journal.pmed.1000268 (PMC2860494; doi:10.1371/journal.pmed.1000268)
Supplement: Table S1 — Participant (factory worker or spouse) characteristics by responder status. (0.02 MB RTF) [file pmed.1000268.s003.rtf]

Table S1: Subject (factory worker or spouse) characteristics by responder status
Characteristics	Responder	Non-responders	No consent	
N
	3537	3565	492	
Male, % (N)
	50.9 (1,800) 	57.7 (2,057)	56.7 (279) 	
Age, mean (SD)
	41.7 (9.2)	41.9 (9.6)	46.2 (7.9)	
Hindu, % (N)
	91.7 (3,243) 	93.8 (3,345)	90.7 (446) 	
Married, % (N)
	97.1 (3,436) 	96.5 (3,439)	97.1 (478) 	
Suffered high blood pressure,
heart disease or stroke, %, (N)	19.3 (684)	14.8 (526)	21.1 (104)	

Currently smoke/chew tobacco, % (N)
	18.3 (646)	20.3 (723)	16.3 (80)	
Hours from factory to place of origin,
Mean (SD) (Quartile 1st; Quartile 3rd)	6.5 (7.0)
(2; 9)	6.8 (8.0)
(1; 9)	8.2 (9.6)
(2; 12)	

Migrant, % (N)
	59.7 (2,112)	60.7 (2,165)	75.6 (372)	
